# Circulating small extracellular vesicles as blood-based biomarkers of muscle health in aging nonhuman primates

**DOI:** 10.1007/s11357-024-01439-y

**Published:** 2024-12-10

**Authors:** Shalini Mishra, Ashish Kumar, Yangen He, Yixin Su, Sangeeta Singh, Mark F. Santos, Rakesh Singh, Jingyun Lee, Cristina M. Furdui, Carol A. Shively, Stephen B. Kritchevsky, Thomas C. Register, Gagan Deep

**Affiliations:** 1 Department of Internal Medicine-Gerontology and Geriatric Medicine, Wake Forest University School of Medicine, Winston-Salem, NC USA; 2 Department of Basic Sciences, College of Osteopathic Medicine, Touro University Nevada, Henderson, NV USA; 3 Atrium Health Wake Forest Baptist Comprehensive Cancer Center, Winston-Salem, NC USA; 4 Department of Internal Medicine, Section On Molecular Medicine, Wake Forest University School of Medicine, Winston-Salem, NC USA; 5 Department of Pathology, Section On Comparative Medicine, Wake Forest University School of Medicine, Winston-Salem, NC USA; 6 Sticht Center for Healthy Aging and Alzheimer’s Prevention, Wake Forest University School of Medicine, Winston-Salem, NC USA; 7 Department of Cancer Biology, Wake Forest University School of Medicine, 575 Patterson Avenue, Biotech Place, 3E-031, Winston-Salem, NC 27101 USA

**Keywords:** Aging, Skeletal muscle, Skeletal muscle-derived small extracellular vesicle, Oxidized protein, MicroRNA

## Abstract

Age-associated loss of muscle mass and function and subsequent mobility decline define poor health outcomes, reduced quality of life, and mortality risk. The rate and extent of aging-related muscle loss varies across older adults. It is challenging to understand the molecular pathogenesis of mobility decline, as anthropometric and imaging techniques, primarily used in muscle function assessment, do not offer much molecular information. Small extracellular vesicles (sEV) are lipid membrane-bound, nano-sized (≤ 200 nm) vesicles which carry a wide array of biomolecules as their cargo. sEV contain cell/tissue-specific signatures on their surface and can be isolated from biofluids. These properties pose sEV as a minimally invasive means to monitor the functional and biological health of difficult-to-access tissues, establishing them as a promising liquid biopsy tool. Here, we first isolated skeletal muscle-derived sEV (sEV^SKM^) from the serum of vervet monkeys (16 to < 25 years old) using alpha sarcoglycan (SGCA) as a muscle-specific sEV surface marker. sEV^SKM^ were extensively characterized for size, concentration, purity, and specificity. Further, sEV^SKM^ isolated from young (11–15 years) and old (25–29 years) monkeys’ serum were characterized for oxidized proteins by mass spectrometry and miRNAs by small-RNAseq. Notably, the analysis of oxidized proteins indicated perturbation of metabolic pathways, actin cytoskeleton, muscle cytoskeleton regulation, and HIF-1 signaling in older monkeys. Furthermore, small-RNAseq analysis identified differential expression of several miRNAs regulating metabolic pathways, inflammation, and stress signaling. Altogether, these results suggest that it is feasible to isolate sEV^SKM^ and use them to identify molecular biomarkers that reflect the physiological state of muscle tissue.

**Supplementary Information:**

The online version contains supplementary material available at 10.1007/s11357-024-01439-y.

## Introduction

In the USA, by 2030, more than 20% of its population will be at 60 years of age [1]. Characteristic human aging phenotypes include the loss of skeletal muscle mass, strength, and function. Longitudinal studies have shown that the muscle mass and muscle strength decline approximately at the age of 75 years. In females, muscle mass and strength decline at a rate of 0.64–0.7% and 2.5–3% per year, respectively [2]. However in males, muscle mass and strength decline rate is 0.8–0.98% and 3–4% per year, respectively [2]. Age-associated muscle mass/function loss is one of the major clinical concerns in the older population as it significantly affects the mobility and functional ability of older adults and reduces their quality of life [3]. Loss of muscle mass and strength leads to variability in gait and balance which increases the probability of falls in older adults [4, 5]. Fall leads to fracture, hospitalization, increased dependence on others, and decreased average life expectancy. Early detection and management of risk factors associated with muscle loss and subsequent mobility decline can help to prevent or delay the onset of mobility decline, improve overall health, quality of life, and reduce healthcare costs.

Currently, various tools are available to assess muscle mass, strength, and performance [6, 7]. Dual-energy X-ray absorptiometry (DXA), magnetic resonance imaging (MRI), computed tomography (CT) scan, bioelectrical impedance (BIA), and peripheral quantitative computerized tomography (pQCT) are the widely used methods to assess muscle mass [6]. These imaging-based methods require specific tools and trained individuals; their high cost and potential radiation exposure limit their utility for many routine clinical applications. Similarly, for muscle function and strength, various performance-based methods, such as short physical performance battery test (SPPB), gait speed, chair rise, grip strength, and accelerometry, are predominantly used [7]. All these methods are simple and feasible, but neither of them accounts for the molecular changes associated with muscle mass/function loss. Despite the paramount use of these techniques in various aging and disease-associated muscle decline studies, both at diagnostic as well as therapeutic levels, they fail to provide molecular information about the muscle tissue [8, 9]. Muscle biopsies are not feasible outside of specific clinical research settings, and repeat measures are especially problematic. Biofluid-based (e.g., blood, urine) assays such as D_3_-creatine dilution methods are sometimes used for assessing muscle mass and function [10, 11]. However, these biofluid-based methods provide limited molecular information and, in most cases, have low specificity as they represent the secretome of the whole body and are not specific to muscle tissue. Thus, despite a great deal of interest, there are still no specific molecular signatures that could predict an individual’s risk of age-related loss of muscle mass and function. One major limiting factor is the lack of methods to repetitively investigate muscle tissue over time to assess molecular changes with aging. Hence, there is an urgent need for novel minimally invasive approaches to assess and monitor muscle aging, as well as early prediction of muscle loss and mobility changes.

Recently, small extracellular vesicles (sEV) have emerged as a promising “liquid biopsy” tool for biomarker/s studies specifically for difficult-to-access tissues/organs [12]. sEV are lipid membrane-bound vesicles (≤ 200 nm in diameter) secreted by cells into the extracellular space. They play a crucial role in inter-cellular communication and cellular homeostasis maintenance [13]. sEV are present in all biofluids and loaded with a vast array of biomolecules (e.g., proteins, nucleic acids, lipids, metabolites) which relate to the cell of origin and represent their physiological/metabolic state [14–16]. sEV have shown potential as novel biomarkers for various diseases due to (a) relative ease of their isolation from biofluids, (b) high stability of their cargo in circulation as they are protected by lipid bimembrane, and (c) feasibility for multi-omics analysis of cargo. In the past decades, various studies have shown the possibility of isolating cell/tissue-specific sEV from biofluids, utilizing the specific sEV surface markers pertinent to the cell/tissue of origin [12, 17, 18]. We and others have reported that various brain cell-derived sEV isolated from the plasma are valuable source of biomarkers for neurodegenerative diseases and in assessment of response to intervention [18–22]. Moreover, we recently showed that highly pure adipose tissue-derived sEV could be isolated from the plasma utilizing specific surface markers and these vesicles in blood reflected the molecular and pathological state of adipose tissue [17]. Guescini et al*.* reported that muscle cells release alpha sarcoglycan (SGCA), a component of the sarcoglycan complex in muscle, positive sEV in plasma [23]. Afterwards, many studies have used SGCA as a surface marker to isolate/characterize the skeletal muscle-derived sEV (sEV^SKM^) from serum and plasma samples [24–26]. In-depth characterization of SGCA-positive sEV shows great promise for assessing muscle-specific health.

Non-human primates (NHPs) are excellent models for many human diseases, owing to their close phylogenetic relationship to humans. Genetically, African green vervet monkeys (*Chlorocebus aethiops*) are 96% homologous to humans. Similar to humans, they develop many age-associated conditions such as cognitive decline, cardiovascular diseases, metabolic disorders (such as diabetes), changes in immune function, and decline in muscle function such as gait speed, climbing, hanging, muscle density, and contractile strength [27–34]. Aging NHPs experience decline in physical function, most evident with decrease in walking speed with age [29], and recent work has demonstrated that slow gait was associated with cognitive deficits 1 year later [35]. Histologic and functional assays of vervet vastus lateralis muscle fibers from old and middle-aged monkeys showed a smaller fiber cross-sectional area and lower force and power-generating ability of fast and hybrid fibers in the older cohort, and these phenotypes were strongly correlated with age [27]. Vervets also experience age-related histologic and functional degenerative changes in the shoulder [32] along with reductions in upper body muscle volume assessed by CT [36]. These characteristics facilitate the study of age-related diseases and biological processes in an NHP model that closely mirrors human aging. In the present study, we isolated sEV^SKM^ from the serum of vervet monkeys using SGCA as muscle-specific sEV surface markers. We extensively characterized sEV^SKM^ for their size, concentration, specificity, and purity by nanoparticle tracking analysis (NTA), flow cytometry, immunogold labeling coupled with transmission electron microscopy (IG-TEM), and super-resolution microscopy. Furthermore, we provided evidence of sEV^SKM^ utility for predicting age-associated muscle function decline by characterizing SGCA-positive sEV from the serum of young (11–15 years) and old (25–29 years) monkeys. Further, we characterized the cargo loaded in sEV^SKM^ to assess various molecular biomarkers. Overall, we focused on isolating and characterizing the sEV^SKM^ from serum, suggesting their usefulness in better understanding the molecular state of muscle.

## Method

### Subjects

In this study, we utilized 24 adult female African green vervet monkeys (*Chlorocebus aethiops sabaeus*) age ranging from 11 to 29 years. Study subjects were born and raised in the vervet research colony (VRC) of Wake Forest School of Medicine. Monkeys were fed standard monkey chow and provided *ad libitum* water. All study procedures were approved by and performed in accordance with the Wake Forest University Institutional Animal Care and Use Committee. Monkey samples (i.e., serum) were divided into 3 groups: characterization, young, and old group. The characterization group was composed of monkeys age ranging from 16 to < 25 years (*n* = 6). The young-age group (*n* = 7) was composed of monkeys from 11 to 15 years, while the old-age group (*n* = 11) consists of 25–29 years-old monkeys.

### Serum collection

For blood collection, monkeys were sedated with ketamine hydrochloride (15 mg/kg intramuscularly) to enable femoral venipuncture. Blood was collected, and serum was isolated using standard procedures; aliquots (200 μL or 500 μL) were immediately frozen at -80 °C until further analysis.

### sEV isolation

To isolate total sEV, ~ 300 μL serum was mixed with 0.1 μm filtered PBS to make up the volume to 500 μL. Serum was then sequentially centrifuged at 500 g for 5 min, 2000 g for 5 min, and 10,000 g for 30 min at 4 °C. Supernatant was filtered with 0.22-micron filter and then incubated with 150 µL of Thromboplastin-D (176065; Fisher Scientific) for 60 min at room temperature (RT). After incubation, 350 μl of 0.1 μm filtered PBS, with protease and phosphatase inhibitor, was added to the samples and centrifuged at 1500 g for 20 min at RT. The supernatant was incubated with 252 μl ExoQuick™ (EXOQ20A-1, System Biosciences) for 1 h at 4 °C followed by centrifugation at 1500 g for 30 min at 4 °C, and the pellet was resuspended in 0.1 μm filtered PBS. To isolate sEV^SKM^, 1 mg of total sEV were incubated with 8 μg of biotin-tagged SGCA (Q16723, Biorybt) and/or muscle specific kinase (MuSK) (PA1-1741, ThermoFisher Scientific) antibody overnight at 4 °C with continuous mixing. Streptavidin-tagged magnetic beads (PI88817; ThermoFisher Scientific) were added to the sample and incubated for 2 h at RT with continuous mixing. Following incubation, beads were washed twice with washing buffer (0.1% TBST) and sEV^SKM^ were eluted from beads by adding 200 µL of IgG elution buffer (21004; ThermoFisher Scientific). Beads were magnetically sequestered, and the supernatant containing the sEV^SKM^ was collected in tubes containing 20 µL of 1 M tris buffer (pH 9).

### Nanoparticle tracking analysis (NTA)

Nanosight NS300 (Malvern Panalytical, UK) equipped with a violet laser (405 nm) and running software version NTA3.4 was utilized for quantification of the hydrodynamic diameter distribution and concentration of sEV. The instrument was primed with PBS (pH 7.4), and the temperature was maintained at 25 °C. For every sample, five videos for 30 s each were recorded. The average of five measurements was plotted to represent the size distribution and concentration (particles/ml).

### Immunogold labeling and transmission electron microscopy (TEM)

CD63 (PA5-92370, ThermoFisher Scientific) antibody was tagged with the 20 nm gold particles following the gold conjugation kit protocol (ab188215, abcam). For immunogold labeling, 200 mesh copper grids (with carbon-coated formvar film) were incubated with 100% ethanol for 20 min to activate the grids. A total of 20 μl sEV samples were incubated with an equal volume of 4% paraformaldehyde for 10 min at RT. Samples were then transferred to grids and incubated for 1 h at RT. Grids were washed with PBS for 3 times (5 min each) and then with 50 mM glycine for 3 times (5 min each). After blocking with 0.5% BSA in PBS for 30 min at RT, grids were incubated with primary antibodies (1:100): CD63 (20 nm gold particle conjugated), SGCA (NBP2-67150, Novus biologicals), MuSK (PA1-1741, ThermoFisher Scientific) for overnight at 4 °C. After washing with 0.5% BSA in PBS (5 min × 3), grids were incubated with appropriate 10 nm gold particle-conjugated secondary antibodies for 2 h at RT in the dark. After washing with 0.1% PBST with 0.5% BSA (5 min × 3), grids were incubated with 2.5% glutaraldehyde for 5 min. Grids were then washed with 0.1% PBST with 0.5% BSA 7 times for 5 min each. Grids were incubated with 1% uranyl acetate for 1 min and then washed with distilled water for 2 min. For negative staining, fixed sEV samples containing grids were incubated with 1% uranyl acetate for 1 min and then washed with water. Grids were imaged using TEM (FEI Tecnai Spirit transmission electron microscope system, Oregon, USA) at reported magnification.

### Immunoblot array

Immunoblot array was prepared in house by immobilizing biotin (positive control), SGCA, and CD63 antibody on the nitrocellulose membrane. Membrane was blocked with 5% non-fat milk in 0.1% TBST. sEV^SKM^ were lysed with RIPA buffer, and all the proteins were biotinylated. After blocking, membrane was incubated with the samples overnight at 4 °C. After washing with 0.1% TBST (3 × 5 min), membrane was incubated with 1:100 diluted horseradish peroxidase (HRP)-avidin for 1 h at RT. Blots were developed with enhanced chemiluminescence ECL reagents.

### Super-resolution microscopy

sEV samples were immunolabeled and imaged using the EV Profiler Kit (#EV-MAN-1.0, ONI) using the direct stochastic optical reconstruction microscopy (dSTORM) technique described in our previous publication [37]. Briefly, sEV were immobilized on microfluidic chips and fixed with the F1 solution (provided in the kit) for 10 min. sEV were then incubated for 50 min with fluorescently labeled antibodies diluted in PBS. The following antibodies were used, either provided in the kit or purchased from a different vendor: CD9-CF488 (kit, excitation (ex)/emission (em): 490/515 nm), CD63-CF568 (kit, ex/em: 562/583 nm), SGCA-AlexaFluor647(sc-27132-AF647, ex/em: 650/671 nm, Santa Cruz), and MuSK (PA1-1741, ex/em: 650/671 nm, ThermoFisher Scientific; labeled with fluorophore in house). Finally, samples were again fixed with F1 for 10 min, and freshly prepared dSTORM-imaging buffer was added before image acquisition. Using the Nanoimager S Mark II microscope (ONI) with 100 × oil-immersion objective, labeled proteins were imaged sequentially at 25, 35, and 50% power for the 640, 561, and 488 nm lasers, respectively, at 1000 frames per channel with the angle of illumination set to 52.5°. Before the start of the imaging session, channel mapping was calibrated using 0.1 μm TetraSpeck beads (#T7279, ThermoFisher Scientific). Subpopulation analyses of sEV expressing one, two, or three markers were analyzed using ONI’s online platform, CODI (https://alto.codi.bio). We performed a density-based clustering analysis with drift correction and filtering to evaluate each vesicle.

### Flow cytometry

Flow cytometry analysis was performed to characterize the surface expression of typical sEV and muscle-specific markers on sEV^SKM^. sEV were labeled with membrane labeling dye CellBrite 488 or 647 (Biotium) with and without the fluorescently labeled antibodies. sEV without dye were used as a control to set the gate for dye-positive events (sEV). sEV labeled with dye but without fluorescent antibodies were used to set the gate for PE/AF647 positive events. Fluorescent antibodies and dye at the same dilution in PBS (filtered through 0.22-micron filter) were also analyzed. We set the cutoff at 2000 on the violet side scatter to exclude the machine background noise. PE- and APC-labeled isotype control were used to confirm the specificity of the fluorescence signals. All samples were acquired on CytoFlex (Beckman Coulter Life Science, Indianapolis, USA) for 60 s at a low flow rate. Filtered PBS was run for 60 s in between the samples. Fluorescent antibodies labeled sEV were also serially diluted and measured for change in mean fluorescent intensity. For further confirmation, 0.25% triton x-100 was added to the sEV, and lysed samples were acquired. Gate applied to detect dye-positive sEV were applied to all other similar samples to confirm the capture of sEV only. The characterization of sEV^SKM^ was performed after labeling with CD63-PE, CD9-AF647, CD81-PE, SGCA-AF-647, MuSK-PE, and GFAP-PE antibodies for 2 h at RT in the dark followed by incubation with membrane labeling dye (CellBrite) at RT for 15 min at 1 × final dye concentration.

### Confocal staining

Streptavidin-tagged agarose beads (15 µL) were incubated with 4 µg of biotin-conjugated SGCA antibody at RT for 1.5 h with frequent mixing in between. A total of 400 µg of total sEV were added to the SGCA antibody labeled beads and incubated overnight at 4 °C on the Hula mixer. Beads were washed with 1% BSA in PBS twice and 2.5 μl fluorescent-tagged SGCA, MuSK, or CD63 antibody was added. Samples were incubated for 2 h at RT in the dark with frequent mixing in between. A total of 100 µL of 1:200 diluted cell Brite membrane dye-AF488 was added into each sample and incubated at RT for 15 min. After washing with 1% BSA in PBS twice, beads were resuspended in 100 μl PBS. A total of 50 μl samples were mounted on the slide and then imaged under Olympus FV1200 SPECTRAL Laser scanning Confocal Microscope (Olympus IX83 inverted platform) at 20 × magnification.

### Oxidized protein analysis by liquid chromatography-mass spectrometry/mass spectrometry (LC–MS/MS)

sEV^SKM^ suspension was lysed in the total reversible oxidation (TRO) lysis buffer (100 mM HEPES, pH 8.5, 8 M urea, 2% SDS) containing protease inhibitor cocktails and 10 mM 4-(5-(Methylsulfonyl)−1H-tetrazol-1-yl)phenol (MSTP). After removal of excess MSTP by chloroform/methanol extraction, protein pellet was reconstituted in 100 µL of TRO lysis buffer. Two microliter of 250 mM Tris(2-carboxyethyl)phosphine (TCEP) was added and incubated at 55 °C for 1 h. Five microliter of 100 mM cystein-reactive phosphate tag (CPT), namely iodoacetamido-LC-phosphonic acid (A52285, ThermoFisher Scientific), was added and incubated at 37 °C for 2 h. Protein was purified using chloroform/methanol extraction, then reconstituted in 200 µL of 50 mM ammonium bicarbonate (ABC) for enzymatic cleavage. Sequencing grade trypsin/lys-C mix was added at the ratio of 1:50 (enzyme to substrate) and incubated overnight at 37 °C. CPT-peptides were enriched using a Fe-NTA Phosphopeptide Enrichment Kit (A32992, ThermoFisher Scientific) according to the manufacturer’s protocol. Enriched peptides were purified using a C18 tip and prepared in 20 µL of 5% acetonitrile with 1% formic acid for LC–MS/MS analysis. Samples were analyzed on an Orbitrap Eclipse Mass Spectrometer (ThermoFisher Scientific, Waltham, MA) coupled with a Vanquish Neo nano-UHPLC system (Thermo Scientific, Waltham, MA) via high FAIMS (field asymmetric waveform ion mobility spectrometry) Pro interface. Peptides were separated on a DNV PepMap Neo (1500 bar, 75 μm × 500 mm) column for 90 min employing linear gradient elution consisting of water (A) and 80% acetonitrile (B), both of which contained 0.1% formic acid. MS2 scans were acquired for peptide identification using a top-speed data-dependent scan where a maximum number of MS/MS spectra were collected from fragmentation of selected precursor ions per 3 s of cycle time between adjacent survey spectra. Dynamic exclusion option was enabled which duration was set to 60 s. To identify proteins, spectra were searched against the UniProt protein FASTA database of human (20,309 annotated entries, Jun 2021) using the Sequest HT search engine with the Proteome Discoverer v2.5 (Thermo Scientific, Waltham, MA). Search parameters were as follows: FT-trap instrument; parent mass error tolerance, 10 ppm; fragment mass error tolerance, 0.6 Da (monoisotopic); enzyme, trypsin (full); # maximum missed cleavages, 2; variable modifications, + 15.995 Da (oxidation) on methionine, and + 221.082 Da (CPT) on cysteine.

### RNA isolation and small RNA-seq analysis

Total RNA was isolated from 100 μl of isolated sEV^SKM^ after RNase treatment, following our published method [18]. Briefly, 450 μl of TRIzol was added to the sEV^SKM^ samples and incubated for 10 min at RT with frequent vortexing in between. Next, 100 μl chloroform was added to the samples and mixed by inverting the tubes, before centrifugation at 14,000 g for 15 min at 4 °C. Clear supernatant was mixed with 2 volume of 100% ethanol and incubated at −20 °C overnight. Samples were then transferred to the RNase mini spin columns (Qiagen, Germantown, MD), and total RNA was isolated as per manufacturer’s recommendation. Total RNA was eluted in 25 μl RNase free water. The concentration of RNA was measured on a nanodrop. Small RNA sequencing using 170 ng of total RNA was performed by LC Sciences (Houston, TX, USA).

### Bioinformatics and statistical analysis

Total oxidized proteins identified from sEV^SKM^ of young and old monkey samples are presented as a Venn diagram (Fig. 3B) using BioVenn software (https://www.biovenn.nl/index.php). A volcano plot, to identify differentially expressed proteins, was prepared by plotting the signal-to-noise ratio (STN) value on the X-axis and -log2(*p* value) on the Y-axis in GraphPad Prism 9 software (GraphPad, San Diego, CA). Associated KEGG pathways were enriched in DAVID software, and bubble graph was prepared in SRplot software. For miRNA analysis, miRNAs present in each group were identified and represented as a Venn diagram utilizing BioVenn software. miRNAs presented in at least 5 samples were sorted, and target analysis was performed in the miEAA (miRNA Enrichment Analysis and Annotation) online platform [38]. KEGG pathways and GO_biological process associated with the targets were enriched using the ShinyGO and g;Profiler online platform [39]. Top enriched pathways and processes were represented as bubble graph utilizing SRplot software. The preparation of bar graphs and statistical analysis was performed by GraphPad Prism 9 software (GraphPad, San Diego, CA). To signify the comparison between groups, as appropriate, a two-tailed, unpaired, Student’s *t*-test was used.

## Result and discussion

### Isolation and characterization of muscle-derived sEV

**Fig. 1:**
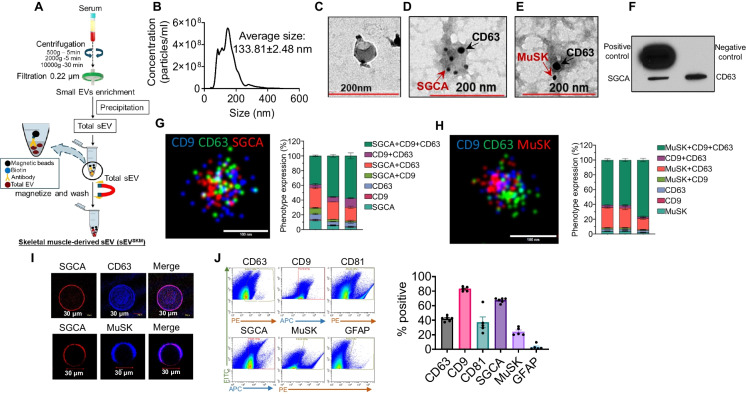
Characterization of sEV^SKM^ isolated from the serum of vervets. **A** Diagrammatic representation of sEV^SKM^ isolation methodology. Total sEV and sEV^SKM^ were isolated from the serum of monkeys (16 to > 25 years old) and characterized by multiple techniques for their purity. **B** Line graph representing size and concentration of sEV^SKM^ (*n* = 6). **C–E** Representative images showing the structure of sEV^SKM^ (**C**), surface expression of SGCA (smaller gold particles—marked by red arrows) and CD63 (larger gold particles—marked by black arrow) (**D**), surface expression of MuSK (smaller gold particles—marked by red arrows) and CD63 (larger gold particles—marked by black arrow) (**E**) by immunogold labeling coupled with transmission electron microscopy. Images were captured at 49,000 × magnification (*n* = 3 each), and scale bar is presented at the bottom of each image. **F** Immunoblot array showing the expression of SGCA and CD63 loaded in sEV^SKM^ (*n* = 1). **G**, **H**. Representative images and bar graphs showing expression of SGCA (**G**) and MuSK (**H**) along with CD63 and CD9 by super-resolution microscopy (*n* = 3). The error bars in the graphical data represent the mean ± SD. **I** Confocal images captured at 20 × , showing expression of SGCA along with CD63 and MuSK on the sEV immobilized on the agarose beads (*n* = 3). **J** Scatter plot and bar graph representing the % abundance of CD63, CD9, CD81, SCGA, MuSK, and GFAP on sEV.^SKM^ surface (*n* = 5–6)

In an effort to further improve purity and efficacy of sEV^SKM^ isolation and characterization, we first sought to identify another potential muscle-specific surface marker besides SGCA. We selected muscle-specific kinase (MuSK), a neuromuscular junction protein, based upon its known high expression in muscle as reported by human protein atlas [40] (https://www.proteinatlas.org/w.proteinatlas.org/) and a prior report suggesting MuSK expression in sEV [41]. We utilized the characterization group (age 16 to < 25 years old) for the isolation of total sEV (*n* = 6). From total sEV, sEV^SKM^ were isolated using either SGCA (sEV^SGCA^) or MuSK (sEV^MuSK^), or both antibodies in combination (sEV^SG/Mu^) by the immune-isolation method (briefly outlined in Fig. 1A).

We first analyzed the total concentration of these sEV by NTA. Interestingly, we observed that SGCA tended to provide a better yield of sEV^SKM^ compared to either MuSK or a combination of both SGCA and MuSK (Supplementary Fig. 1A), though this difference was not statistically significant. Further, we used flow cytometry to assess the surface expression of CD63, a known exosome-related tetraspanin biomarker, on these sEV. Notably, we did not observe any statistically significant difference in CD63 expression between all the three approaches (Supplementary Fig. 1B). Taken together, our data suggested that SGCA alone allows a comparable yield of SEV^SKM^ from biofluid without compromising the purity. Furthermore, sEV^SGCA^ also showed expression of MuSK on their surface, as confirmed using the immunogold method (Supplementary Fig. 1C), which suggested that, although MuSK inclusion did not further improve the yield or purity of sEV^SKM^, it could serve as a secondary validation marker to assess the purity of isolated sEV^SKM^. Since we did not observe any advantage of using multiple markers to isolate sEV^SKM^ in terms of yield and purity, a single marker SGCA was used for the isolation of sEV^SKM^.

While SGCA-positive sEV in circulation have been previously reported [23–26], a few studies using less sensitive western blot-based methods failed to detect SGCA-positive sEV in the plasma/serum total sEV pool [42, 43]. In this study, we performed extensive characterization of sEV^SKM^ using multiple high-sensitivity methods (Fig. 1). After enrichment from total sEV, utilizing an immune-isolation method using SGCA antibody, sEV^SKM^ were first characterized for their size and concentration using NTA which confirmed that their average size (133.81 ± 2.48 nm) is in the acceptable size limit for sEV (≤ 200 nm) (Fig. 1B). Next, transmission electron microscopy (TEM) confirmed the double membrane-bound structure with a typical size of sEV (Fig. 1C). We then utilized a novel approach of double labeling of sEV with two different-sized gold particle-tagged antibodies (10 nm or 20 nm) and confirmed the surface co-expression of SGCA (Fig. 1D) and MuSK (Fig. 1E) with CD63. Next, we used antibody array to validate the presence of SGCA along with CD63 loaded in sEV^SKM^ (Fig. 1F).

Super-resolution microscopy is a powerful microscopy tool which allows imaging sEV at a single molecular level. Super-resolution microscopy also confirmed the presence of the SGCA and MuSK on the sEV^SKM^ along with exosomal markers CD63 and CD9 (Fig. 1G and H). Interestingly, we observed most of the sEV^SKM^ were positive for SGCA, CD63, and CD9 (Fig. 1G, bar graph) and MuSK, CD63, and CD9 (Fig. 1H, bar graph), confirming the purity and muscle specificity of these vesicles.

Immunofluorescence staining of sEV immobilized on agarose beads, using fluorescent antibodies against CD63 and SGCA, simultaneously confirmed co-localization of CD63 and SGCA on sEV^SKM^ (Fig. 1I; top panel). Similarly, surface co-expression of SGCA with a second muscle-specific marker MuSK on the beads was also confirmed (Fig. 1I; bottom panel). Next, nano-flow cytometry also showed the abundance of skeletal muscle marker SGCA and MuSK along with exosomal surface markers (CD63, CD9, and CD81) on sEV^SKM^ (Fig. 1J). Since total sEV is a heterogenous pool of various cell/tissue-derived sEV, thus the possibility of contamination of other cell/tissue-specific sEV while pulling down a specific population is likely. Thus, we also checked the presence of GFAP-positive sEV (an astrocyte-derived sEV marker) in sEV^SKM^ as a negative marker. Importantly, the negligible presence of GFAP on sEV^SKM^ further confirmed the purity of isolated sEV^SKM^ (Fig. 1J). Altogether, our data suggested that skeletal muscle-derived sEV are present in serum and can be isolated from the blood using SGCA with high purity.

### Characterization of sEV^SKM^ for age-associated pathology in young and old vervet monkeys

**Fig. 2:**
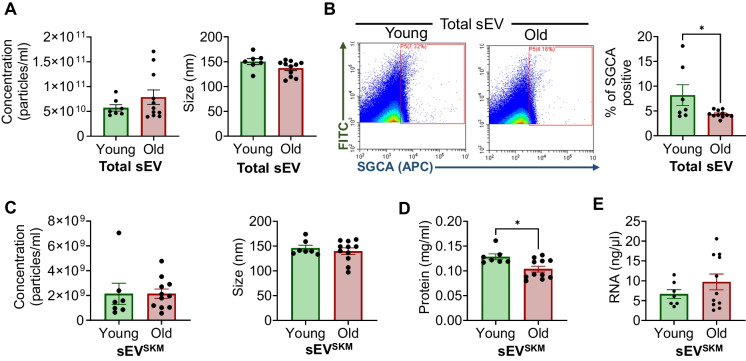
Characterization of serum sEV^SKM^ in young and old vervets. Total sEV and sEV^SKM^ were isolated from the serum of young (≤ 15 years) and old (≥ 25 years) vervets and analyzed. **A** Bar graphs represent the average concentration and size of total sEV. **B** Representative scatter plots and bar graphs showing the percentage abundance (mean ± SEM) of SGCA positive sEV in total sEV of young and old vervet. **C** Bar graphs representing the average sEV^SKM^ concentration (left panel) and size (right panel) for young and old vervets. **D**, **E** Bar graphs representing the total protein (**D**) and RNA (**E**) content in sEV^SKM^ of young and old vervets (*n* = 7–11). *p ≤ 0.05

To establish the translation significance of serum-derived sEV^SKM^, we isolated total sEV followed by sEV^SKM^ separation from the serum of young (11–15 years; *n* = 7) and old (25–29 years; *n* = 11) vervet monkeys following the method described above (Fig. 1A). We first characterized the total sEV by analyzing changes in their secretion profile employing NTA and did not find any significant age-related differences in the total concentration or size of total sEV (Fig. 2A). We next characterized total sEV for percent abundance of SGCA positive sEV (sEV^SKM^) between young and old vervets using flow cytometry. We observed significant decrease in sEV^SKM^ in the old vervets (Fig. 2B). Since it is well established that muscle mass decreases with age, we speculate that this reduction in the sEV^SKM^ is likely attributable to the overall loss of muscle mass. However, since sEV from muscle cells are also known to promote the differentiation and proliferation of myoblasts [44, 45], it is also plausible that a decrease in sEV^SKM^ secretion may play an active role in exacerbating muscle loss, highlighting the potential of sEV^SKM^ as both, a marker and modulator of muscle health.

Next, we isolated sEV^SKM^ from the total sEV and characterized them for the concentration and size using NTA. Even though we observed a significantly lower SGCA-positive population in total sEV in old vervets by flow cytometry (Fig. 2B), we did not see a significant difference in size or concentration of sEV^SKM^ after immune-isolation (Fig. 2C), possibly due to the known limited efficiency of the immune-isolation method.

We further analyzed the protein and total RNA cargo of sEV^SKM^ between young and old vervets (Fig. 2D). We observed a significant decrease in protein cargo in old vervets compared to young, while total RNA content remains unchanged (Fig. 2D). Taken together, these findings suggest that aging disrupts not only the secretion of sEV^SKM^ secretion but could also impact their cargo composition.

### Characterization of sEV^SKM^ protein cargo for age-associated pathology in young and old vervets

**Fig. 3:**
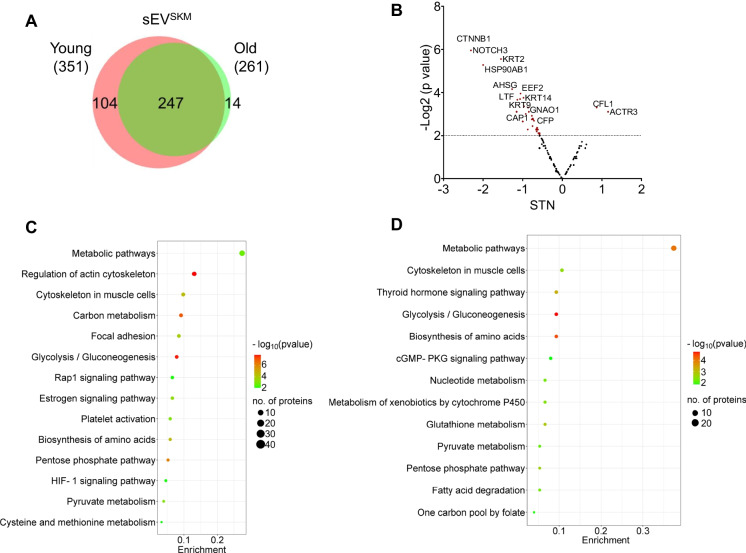
Characterization of oxidized proteins in sEV^SKM^ from young and old vervets. **A** Venn diagram representing the number of oxidized proteins identified in sEV^SKM^ of young and old vervets (*n* = 3/group, 6 samples were randomly pooled into 3 separate samples). **B** Volcano plot representing the significantly perturbed oxidized proteins in sEV^SKM^ of young and old vervets. **C** Bubble plot representing KEGG pathways associated with the significantly perturbed (STN ≤ − 0.3 and ≥ 0.3) oxidized proteins in sEV^SKM^ of young and old vervets. **D** Bubble plot representing KEGG pathways associated with the oxidized proteins uniquely present in sEV^SKM^ of young vervets

Oxidative stress is one of the key underlying factors in age-associated muscle mass/function decline [46, 47]. Due to increased oxidative stress, reactive oxygen species (ROS) accumulation causes protein oxidation leading to subsequent muscle loss. Thus, we next measure the oxidized protein content in sEV^SKM^. To measure total reversible oxidation (TRO), we employed cysteine-reactive phosphate tag (CPT) to enrich peptides containing cysteine with a wide range of oxidation states including sulfenylation, intra-, and inter-molecular disulfide, glutathionylation, hydropersulfidation, and S-nitrosation, and peptides were identified by LC–MS/MS analysis [48]. We identified 351 and 261 oxidized proteins in sEV^SKM^ of young and old monkeys, respectively (Fig. 3A) representing a decrease in the number of oxidized proteins in the sEV^SKM^ from old vervets. With aging, protein diversity could change due to altered rate of protein translation or disrupted protein homeostasis processes such as protein folding and/or protein complexes formation [49]. In concord with these age-related changes, we detected a similar trend in sEV^SKM^, both at total protein concentration (Fig. 2D) and oxidized proteins number (Fig. 3A). These findings suggest that sEV^SKM^ cargo could reflect the broader age-related changes in muscle cell.

Next, we identified significantly perturbed proteins in sEV^SKM^ from young and old vervets (Fig. 3B). We observed that cofilin 1 (CFL1) and actin-related protein 3 (ACTR3) were the most upregulated oxidized proteins in sEV^SKM^ of old-aged monkeys compared to young-aged monkeys. Both proteins are part of the actin polymerization/depolymerization complex which regulates muscle cell contraction and force generation [50, 51] suggesting a shift in cytoskeletal dynamics with aging. Furthermore, we observed catenin beta 1 (CTNNB1), NOTCH3, heat shock protein 90 alpha class B (HSP90AB1), alpha-2-HS-glycoprotein (AHSG), and eukaryotic elongation factor 2 (EEF2) were significantly downregulated oxidized proteins in sEV^SKM^ of old-aged monkeys compared to their young counterpart. CTNNB1 and AHSG regulate the glucose uptake in muscle cells [52, 53], thus facilitating the muscle energy metabolism. EEF2 and HSP90AB1 play important roles in protein translation and folding processes [54], and NOTCH3 is reported to regulate muscle proliferation [55] contributing to muscle growth and regeneration, all essential for preserving muscle mass. These findings indicate that aging is associated with disruptions in pathways related to muscle metabolism, actin cytoskeleton dynamics, and protein homeostasis, all of which likely contribute to age-related muscle loss. In this regard, Day et. al. recently evaluated biopsies of vastus lateralis muscle in the participants of Study of Muscle, Mobility and Aging (SOMMA) and reported that increased oxidation of key proteins involved in muscle structure and contraction was negatively correlated with the muscle function [56]. Specifically, increased oxidized levels of sarcomeric proteins myomesin-1, myomesin-2, and nebulin were associated with slower walking speed, and increased oxidation of myomesin-2, alpha-actinin-2, and skeletal muscle alpha-actin were associated with lower leg power and strength [56]. This study confirmed that levels of these oxidized proteins in muscle were directly associated with compromised muscle health [56]. In the present study, there was a relatively lower prevalence of oxidized proteins in sEV^SKM^ in old monkeys, perhaps due to the retention of oxidized proteins within the muscle of older monkeys. Taken together, these findings suggest that sEV^SKM^ could play a critical role in the clearance of oxidized proteins within the muscle, thereby protecting the cells from oxidative damage. However, aging may impair this clearance mechanism, leading to the accumulation of oxidized proteins, causing muscle damage. It is important to determine if there is an inverse relationship between oxidized protein content within the muscle vs. exported in the sEV^SKM^, and ongoing and future studies will provide answers to that and other critical questions.

Next, we analyzed 247 shared proteins in sEV^SKM^ from young and old vervets and identified the significantly altered proteins (STN ≤ − 0.3 and ≥ 0.3). KEGG pathways associated with these perturbed proteins were enriched by the DAVID online tool. We observed several pathways including metabolic pathway, regulation of actin cytoskeleton, cytoskeleton in muscle cells, Rap 1 signaling pathway, biosynthesis of amino acids, HIF-1 signaling, and pyruvate metabolism as the top-hit pathways. In our analysis for oxidized proteins, we found 104 proteins which were detected only in sEV^SKM^ from young vervets. KEGG pathway enrichment of these proteins showed several pathways including metabolic pathway, thyroid hormone signaling, cGMP-PKG signaling, nucleotide metabolism, glutathione metabolism, fatty acid degradation, and one carbon pool by folate (Fig. 3D).

These findings indicate that aging could perturb core metabolic and cytoskeletal regulatory pathways as well as critical signaling and metabolic functions. These age-related alterations in sEV^SKM^ proteins present as potential novel targets for diagnostic as well as therapeutic intervention for mitigating muscle mass/function loss in the elderly. However, further studies are needed to validate these potential targets in corresponding muscle tissues.

### Characterization of sEV^SKM^ miRNA cargo for age-associated pathology in young and old vervets

**Fig. 4:**
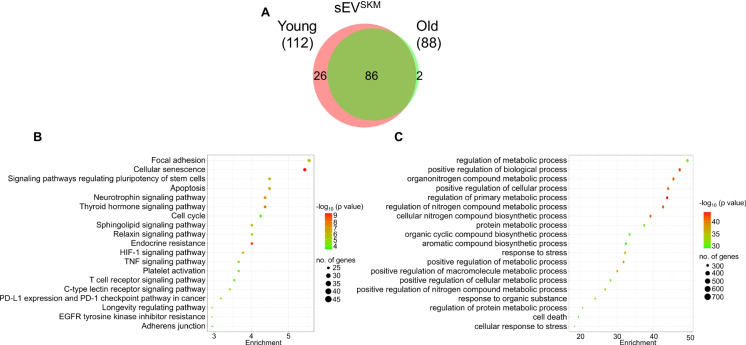
Characterization of miRNAs in sEV^SKM^ from young and old vervets.** A** Venn diagram representing the number of miRNAs identified in sEV^SKM^ from young and old vervets (*n* = 3/group, 6 samples were randomly pooled into 3 separate samples). **B**, **C**. Bubble plot representing the top significantly enriched KEGG pathways (**B**) and Gene ontology-Biological process (**C**), associated with perturbed miRNAs

We next analyzed the miRNA cargo loaded in the sEV^SKM^ of young and old vervets and identified 112 and 88 miRNAs in sEV^SKM^ of young and old vervets, respectively (Fig. 4A). Similar to protein cargo, here also we observed low diversity of miRNA in sEV^SKM^ from old vervets. The biological pathways associated with the most differentially expressed miRNAs were identified using miEAA and ShinyGO tools. Analysis of KEGG pathways associated with the miRNA targets showed that top enriched pathways included focal adhesion, cellular senescence, apoptosis, neurotrophin signaling, cell cycle, relaxin signaling, endocrine resistance, HIF-1 signaling, TNF signaling pathway, T-cell receptor pathways, and adherence junctions (Fig. 4B). Further enrichment for biological processes showed that the most significantly associated biological events were related to metabolic regulation, stress response, and cell death (Fig. 4C).

Both oxidized protein and miRNA analyses of sEV^SKM^ consistently identified the involvement of three primary biological processes: metabolic pathways, cell–matrix interactions (actin cytoskeleton, muscle cytoskeleton, adherence junction, focal adhesion), and inflammatory responses. These pathways are known to be involved in age-related muscle mass and function loss. In aging, metabolic changes are considered to be a significant contributor to muscle mass loss [57, 58]. These changes include imbalances in protein synthesis/degradation and impaired glucose metabolism. Skeletal muscle is a key site for glucose uptake where it is actively converted into glycogen [59] serving as a local energy substrate during exercise. Metabolically, muscle is a very flexible organ which rapidly adapts to use glucose, fatty acid, or amino acids as their substrate. Flexibility of skeletal muscle to use lipid or carbohydrate as substrate is associated with different panels of structural proteins [60]. Mitochondrial dynamics also play a critical role in metabolic adaptation [61, 62]. In this study, we found significant enrichment of HIF-1 signaling at both the protein and miRNA levels. HIF 1, a master intracellular oxygen sensor, regulates mitochondrial biogenesis, respiratory chain, TCA cycle, and membrane potential [63]. Further, low-grade persistent chronic inflammation is a commonly observed aging phenotype. The presence of damaged macromolecules, cellular and immune senescence, and increased activation of the coagulation system are some of the possible sources/mechanisms of chronic inflammation in the aging population [64, 65]. Inflammation induces muscle function/mass loss by promoting protein degradation, inhibiting protein synthesis, and activation of satellite cells [66, 67]. Importantly, in peripheral circulation, sEV^SKM^ cargo provided a window into these major metabolic, cytoskeleton, and inflammation-related molecular changes in the skeletal muscle of old vervets.

This study delineates significant advancement in skeletal muscle-derived sEV characterization, including the isolation and purity validation of sEV^SKM^, utilization of an excellent non-human primate model for aging (a close phylogenic neighbor of humans), and identification of molecular changes with aging. Limitations of this study include the need to validate identified molecular changes in corresponding muscle tissues and the observed molecular changes in sEV^SKM^ by other methods and in multiple possible models. Despite these weaknesses, the present study is a major advancement in the isolation of sEV^SKM^ from serum, validating their purity by multiple methods and identifying potential molecular changes in muscle with aging. Ongoing studies are evaluating these alterations in blood sEV^SKM^ in relation to skeletal muscle biopsy and physical and functional phenotypes in these same subjects.

## Supplementary Information

Below is the link to the electronic supplementary material.

Supplementary file1 (PDF 188 KB)
