# Supplementary material for: Circulating small extracellular vesicles as blood-based biomarkers of muscle health in aging nonhuman primates
Source: GeroScience. 2024 Dec 10;47(3):3709–23. doi: 10.1007/s11357-024-01439-y (PMC12181483; doi:10.1007/s11357-024-01439-y)
Supplement: Supplementary file 1 — Supplementary file1 (PDF 188 KB) [file 11357_2024_1439_MOESM1_ESM.pdf]

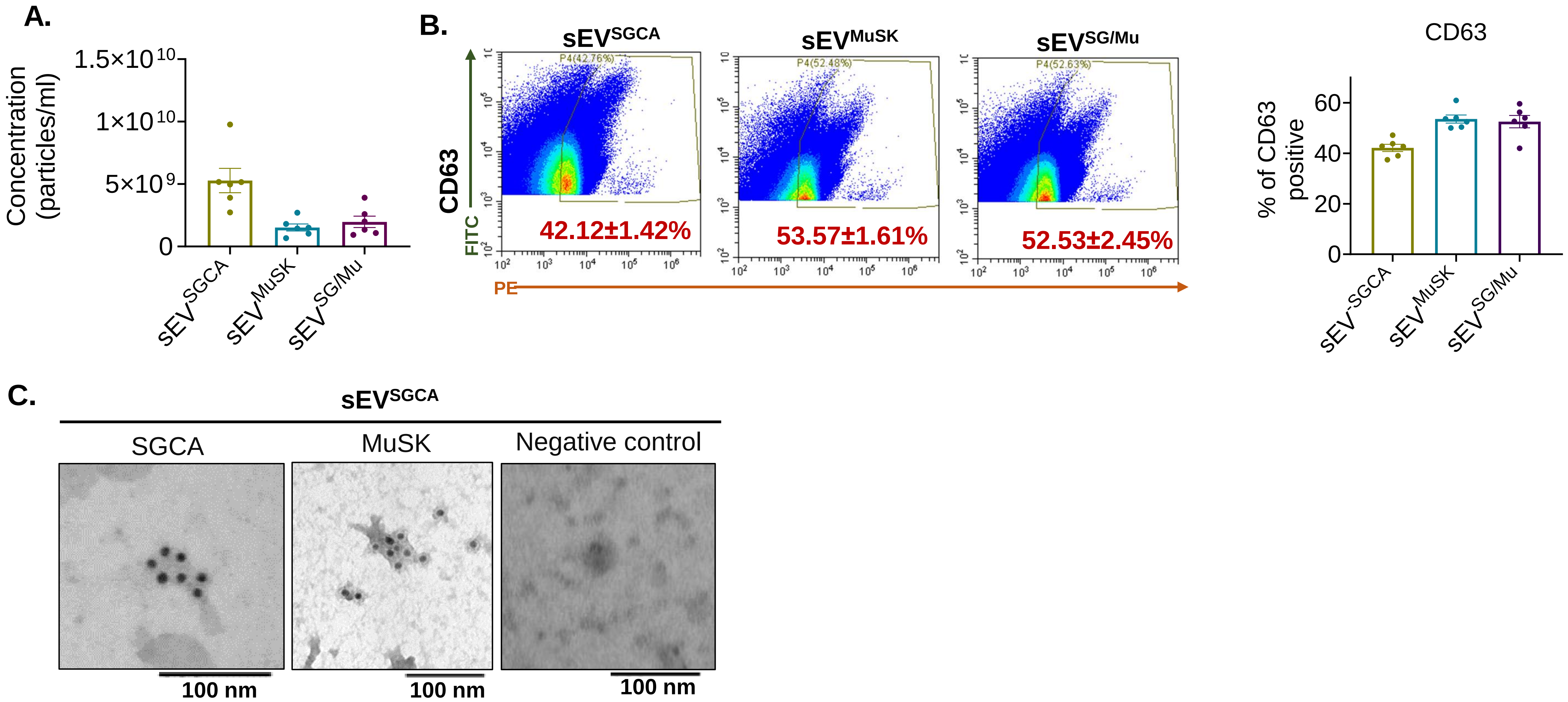

Supplementary figure 1. Comparison of different method of sEV^{SKM} isolation. sEV^{SKM} were isolated either by utilizing biotin tagged SGCA antibody, or by biotin tagged MuSK antibody or by utilizing them in combination. **A.** Bar graph represented the concentration of sEV^{SKM} isolated by all the methods (n=6). **B.** Scattered plot and bar graph represent the percentage abundance of CD63 positive sEV. Number on the scatter plot represent the mean±SEM values. (n=6). **C.** Representative images (98,000X magnification, scale bar at the bottom of images) are presented showing the expression of SGCA and MuSK on the surface of SGCA positive sEV as analyzed by immunogold labeling/TEM. (n=3).
